# Wheat *Fusarium* Protease Specificity and Effect on Dough Properties

**DOI:** 10.3390/foods10071585

**Published:** 2021-07-07

**Authors:** Ray Bacala, Bin Xiao Fu, Katherine Cordova, Dave W. Hatcher

**Affiliations:** Canadian Grain Commission, Winnipeg, MB R3C 3G8, Canada; ray.bacala@grainscanada.gc.ca (R.B.); katherine.cordova@grainscanada.gc.ca (K.C.); hatcherdw@gmail.com (D.W.H.)

**Keywords:** *Fusarium*, wheat, protease, dough properties

## Abstract

*Fusarium* infection is a worldwide agricultural problem of billion dollar proportions globally, and it has increasingly threatened entire regional food supplies. In addition to the toxin deoxynivalenol (DON), *Fusarium* species express digestive enzymes that degrade starch and protein, affecting the quality of infected grains, especially wheat processing performance which depends largely on gluten proteins. In this study, the impact of *Fusarium* protease on the functionality of Canada Western Red Spring (CWRS) wheat was assessed by adding *Fusarium*-damaged kernels (FDK) to a FDK-free base wheat sample. Digestion of beta-casein by extracts of flours, milled from sound and FDK-spiked wheat samples, demonstrated elevated cleavage in FDK-spiked flour extracts as follows: *N*-terminal to lysine (eight-fold), *N*- and *C*-terminal to isoleucine (four-fold and three-fold, respectively), *N*-terminal to tyrosine (three-fold) and *C*-terminal to arginine at P1′ (five-fold). Comparison of abbreviated (45 min) and standard (135 min) extensigraph test results indicated that desirable increases in dough resistance to extension (R_max_) due to gluten re-polymerization after longer resting were partially to completely counteracted in FDK-spiked flours in a dose-dependent manner. Baking tests confirmed that while loaf volume is similar, proofed dough from FDK-spiked samples caused detectable loaf collapse at 3% FDK. Extensigraph R_max_ and *Fusarium* protease levels were inversely related, and effected by both the extent and severity of infection. While the current FDK tolerances for grading Canadian wheat can effectively control protease damage, prevalence of deoxynivalenol (DON) weak- and non-producing *Fusarium* strains/species (e.g., *F. avenaceum*) in some growing regions must be considered to protect functionality if grading is solely based on DON content.

## 1. Introduction

*Fusarium* infection in wheat is a global problem involving a large number of *Fusarium* species, each with a different prevalence in different parts of the world. In addition to the severe economic loss and potential threat to regional food supplies, some of these species are also capable of causing fusariosis in immunocompromised humans; a treatment-resistant infection that is rare but globally on the rise [1]. In addition to the toxin deoxynivalenol (DON), *Fusarium* species also produce degradative enzymes that aid initial infection and provide sustained nourishment for the established infection. Proteomic analysis of the extracellular proteins (exoproteome) produced by *Fusarium graminearum* cultured with plant cell wall isolates revealed *F. graminearum* produced all required digestive enzymes for complete cell wall degradation [2]. These enzymes include both polysaccharide-metabolizing enzymes and proteases, which are primarily involved with initial infection [3]. While DON production typically increases several days after infection, production of secreted proteases and polysaccharide-digesting enzymes peaks within 2 days of infection [3].

*Fusarium* infection reduces wheat end-use quality [4,5,6,7,8,9,10,11,12,13], presumably by impairing seed maturation and digestion of starch and protein for sustenance. *Fusarium* proteases remain dormant in harvested seed but reactivate during dough making, thus affecting dough handling properties and baking performance [14,15,16,17]. While a large amount of work has been carried out in this field, most research has been based on non-specific tests [3,15,18] or proteomic/genomic identification of gene sequences [19]. Additionally, very little work has been carried out on identifying the specificities of individual proteases or their abilities in damaging gluten proteins. Two proteases have been purified and characterized from *F. culmorum* grown on a wheat gluten-containing culture; a subtilisin-like serine protease with maximum activity at pH 8.3–9.6 [20] and a trypsin-like protease with maximum activity at pH 9.0 [21]. While these two proteases were highly similar to genes in other fungal species, a combined genetic and proteomic analysis has identified up to 134 secreted peptidases/proteases in *F. graminearum*, forming several redundant groups [19]. Culturing experiments have also shown that the nutrient composition of the medium influences expression of secretory enzymes. For example, *F. graminearum* releases a host of excretory proteins when grown on cell wall, but only four when grown on glucose alone [2]. Pekkarinen et al. [18] observed no protease activity for three *Fusarium* species grown on mineral media until glucose was depleted and proteases active between pH 6–9 on gluten or barley grain medium. Additionally, *Fusarium poae* produced an acid protease with an optimal pH between 3.0 and 3.5 when grown on barley grains and in liquid media containing wheat gluten protein [18]. Therefore, understanding the specificities of gluten-degrading proteases and how they may differentially present on host species is essential to the development of an assay sensitive and specific enough to detect *Fusarium* protease at levels where declines in dough properties and baking performance are observed.

Measurement of *Fusarium* protease is important as, with DON production, protease activity differs between *Fusarium* spp. [17] and across strains within a species [3]. While protease and DON are likely both positively correlated with the degree of infection, DON levels will not necessarily predict protease content or loss of baking quality [10]. Kreuzberger et al. [10] also concluded that there was no significant impact on technological quality as long as DON was within EU-mandated tolerances. This assertion, however, requires the knowledge that all species and strains present in a growing region actually produce DON. For example, *F. avenaceum* cannot produce DON as the pathway is genetically inactivated [22], but is prevalent (though declining) in parts of Western Canada [23], Eastern Canada [24] and among the most prevalent *Fusarium* species in six European countries [25]. The actual or potential existence of non- or weak-DON producing *Fusarium* species/strains in any one growing region makes drawing conclusions on damage from *Fusarium* protease on functionality solely from DON levels a potentially erroneous assumption.

The objectives of this study were to characterize the prevailing *Fusarium*-specific protease specificity in infected wheat and detect elevated protease activity in blends of infected kernels at levels where technological quality (extensigraph R_max_) was significantly reduced. This work will also form the basis for ensuring any loss of functionality due to protease damage remains controlled in all growing regions if any changes are made to grading tolerance levels for *Fusarium* damage. This will enable screening of *Fusarium* species/strains and variation over growing seasons to understand how protease levels relate to technological quality, pathogenicity and various environmental stresses.

## 2. Materials and Methods

### 2.1. Seed Samples

Bread wheat (cv. AAC Brandon), graded as No. 1 Canada Western Red Spring (CWRS) and free from *Fusarium* infection, was used as a base for blends spiked with *Fusarium*-damaged kernels (FDK). FDKs were handpicked by trained Canadian Grain Commission (CGC) inspectors from *Fusarium*-infected samples into three categories based on infection severity: mycelia (FDK-M; presence of mycelia beyond the seed crease, but otherwise sound), affected (FDK-A; close to normal kernel weight and shape with chalky seed coat) and distorted (FDK-D; reduced seed size, thin seeds with chalky seed coat).

### 2.2. Extensigraph and Baking Test

Wheat blends were prepared by adding FDK to the clean base by mass as follows: FDK-M (0, 5 and 10%), FDK-A (0, 0.25, 0.8, 1.5 and 3%) and FDK-D (0, 0.25, 0.8, 1.5 and 3%). Wheat samples were tempered and milled following the AACC International Method 26-21.02 [26] on a Buhler test mill to generate flour at a constant extraction rate of 74%. Dough strength was measured in duplicate by extensigraph following AACC method 54-10.01 [27]. For each replicate, two 150 g pieces of dough were prepared, rounded and molded into cylindrical shape and rested in a humidified chamber of the extensigraph for 45 min prior to first stretching. The stretched dough was reshaped and stretched again after a second resting period of 45 min, followed with a final stretching after a third 45 min rest. The R_max_ values (BU) of the first and third stretches (45 and 135 min) were recorded; 135 min is the standard test time [27]. To demonstrate the impact of FDK on baking quality, duplicate loaves were prepared using flour from sound base wheat, sound base +3% FDK-A and sound base +3% FDK-D were prepared using the lean no time (LNT) baking method [28]. The LNT bake test eliminates the use of an oxidant and reduces the salt and shortening levels to 1%. It better reflects the impact of dough properties on loaf characteristics compared to other test bake methods [28]. A new objective parameter, loaf top ratio, was introduced to qualify the handling properties [28]. The crumb images were captured with loaf slices using C-Cell instrument (Calibre Control International Ltd., Warrington, UK).

### 2.3. Fusarium Protease Assay

Wheat blends of clean base spiked with FDK-D at 0, 0.25, 0.4, 0.5, 0.8, 1.0, 1.25, 1.5, 2, 2.5 and 3% were prepared by mass and ground with Perten mill 3100. The ground whole meal (0.1 g) was extracted with ammonium bicarbonate buffer (150 mM, pH 7.8, 1000 μL) by orbital mixing for 15 min at room temperature. Extracts were centrifuged at 14,000× *g* for 5 min, filtered through a 1.0 μM glass fiber syringe filter and diluted 40-fold in ammonium carbonate buffer. Protease activity was assayed within one hour of preparation using the Förster Resonance Energy Transfer (FRET)-paired peptide substrate Mca-R-P-K-V-E-Nval-W-R-K(Dnp)-NH2 (Peptide ES-002, R&D Systems, Minneapolis, MN, USA). Substrate (1 mg) was dissolved in AMBIC (4.7 mL), stored in 500 μL aliquots protected from light and only thawed once. Diluted extracts (90 μL) were delivered to 96-well black-walled, clear-bottomed microtitre plates (3 wells per extract) and the assay initiated by the addition of substrate solution (10 μL) using a multi-channel pipette. Fluorescence (excitation 320 nm, emission 420 nm, no filter) was measured every 5 min for 60 min at 37 °C. Initial reaction rates were calculated for each time point and the slope of the plot of initial rates as a function of time used as the activity for each sample. Five replicate analyses were conducted.

### 2.4. Single Seed Protease Assay

FDK-A and FDK-D were handpicked from samples obtained from six individual *Fusarium*-infected samples from different sites across Western Canada in the 2018 crop year. From each site, 10 FDK-A and FDK-D seeds were weighed and ground using an Omni Bead Ruptor Elite (Kennesaw, GA, USA) in 2 mL tubes with a single 6.5 mm ceramic bead for 30 s at 6.0 m/s. Ammonium bicarbonate buffer (150 mM, pH 7.9, 1000 μL) was added to each tube and samples were extracted at room temperature on an orbital mixer. Extracts were decanted into a 1 mL syringe, filtered through a 1 μM glass fiber filter and serially diluted 2000-fold in the same buffer. Protease activity was assayed as described above for whole meal samples.

### 2.5. Verification of Protease Specificity

Enzyme extracts were prepared from ground samples (200 mg) of clean base and clean base spiked with 3% FDK-D with ammonium bicarbonate, 150 mM, pH 7.8 (1.5 mL) at room temperature by sonication (1 min), followed by 60 min using an orbital mixer. Extracts were microfuged and filtered through a 1 μM glass fiber syringe filter (enzyme extracts). Bovine beta-casein (1 mg/mL) was prepared in 50% n-propanol containing 1% (*w*/*v*) dithiothreitol, aliquotted to microfuge tubes (600 μL) and denatured by incubation at 60 °C for 15 min. Protein was precipitated with two volumes of 20% trichloroacetic acid, followed by immediate centrifugation and acetone washes (2 × 2 mL). The pellets were allowed to air dry and were then reconstituted in 100 μL of aqueous 1 mg/mL Rapigest (Waters, Milford, NH, USA) by vortex mixing and sonication. Ammonium bicarbonate, 150 mM, pH 7.8 (300 μL) was added plus 100 μL enzyme extract (clean base, 3% FDK-D or buffer for the digestion blank). Samples were incubated overnight at 37 °C, acidified using formic acid (5 μL) and further incubation for 30 min at 60 °C to destroy the Rapigest detergent. Digests were filtered through SINGLEStEP eXtreme filter vials (PTFE, 0.45 μM, Thomson Instrument Company, Oceanside, CA, USA) and subjected to peptide mapping by LC-MS on a Waters (Milford, NH, USA) G2-Synapt mass spectrometer as previously described [29]. Briefly, an Agilent (Wilmington, NC, USA) Zorbax SB300-C8 column (2.1 × 100 mm, 1.8 μM particle size, 300 Å pore size) was used with a water/acetonitrile (ACN) plus 0.1% formic acid gradient at 0.25 mL/min and 50 °C from 5 to 60% ACN over 50 min. The mass spectrometer was operated in ESI positive mode using MS^E^ with a 0.5 s scan time.

Peptides were matched against the bovine beta-casein mature protein sequence (UNIPROT accession P02666, residues 16–224) using the ion accounting function of Progenesis QIP (Waters, Milford, NH, USA) with non-specific cleavage. Due to the potential activity of multiple endoproteases and further processing of peptides by exopeptidases present in the sample, all matched peptides were considered equally for determining the protease specificity of the sound and FDK-D extracts.

## 3. Results

### 3.1. Impact on Dough Strength

**Figure 1:**
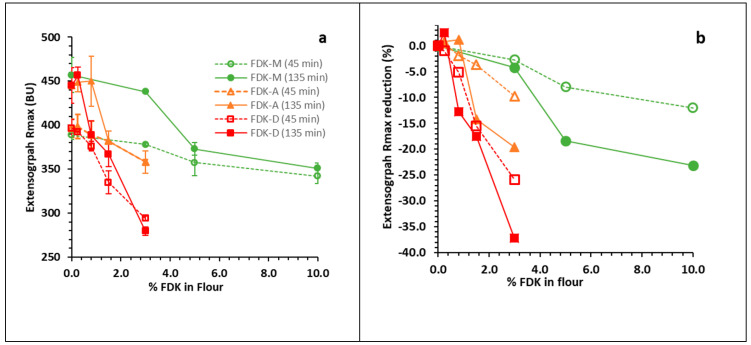
Absolute (**a**) and relative (**b**) reduction of Extensograph Rmax between 45 min (abbreviated) and 135 min (standard) tests with increasing FDK content. Rmax typically increases between 45 min and 135 min ((**a**), broken versus solid lines) as re-polymerization occurs during dough development, increasing resistance and decreasing extensibility. Protease damage is evident by the convergence of the broken (45 min) and solid (135 min) lines, indicating reduced increase in Rmax with increasing Rmax.

The extensigraph R_max_ (135 min) of the flour samples milled from the wheat blends (Figure 1a, solid lines) decreased from sound base (445 ± 20 BU SD for FDK-A and FDK-D, 457 ± 20 BU for FDK-M) significant enough to indicate a loss of dough strength at 0.8% FDK-D (389 ± 16 BU), 1.5% FDK-A (382 ± 20 BU) and 5% FDK-M (351 ± 6 BU). During dough resting in the extensigraph test, gluten re-polymerization continues from the first stretch at 45 min to the third stretch at 135 min. This leads to increased resistance (elasticity) and decreased extensibility, as reflected by higher R_max_ values in the 135 min stretch than the 45 min stretch (Figure 1a, solid versus broken lines). In order to compare the impact of FDK content on R_max_ more directly, data were also expressed as the percent loss from sound flour for each series (Figure 1b).

**Figure 2:**
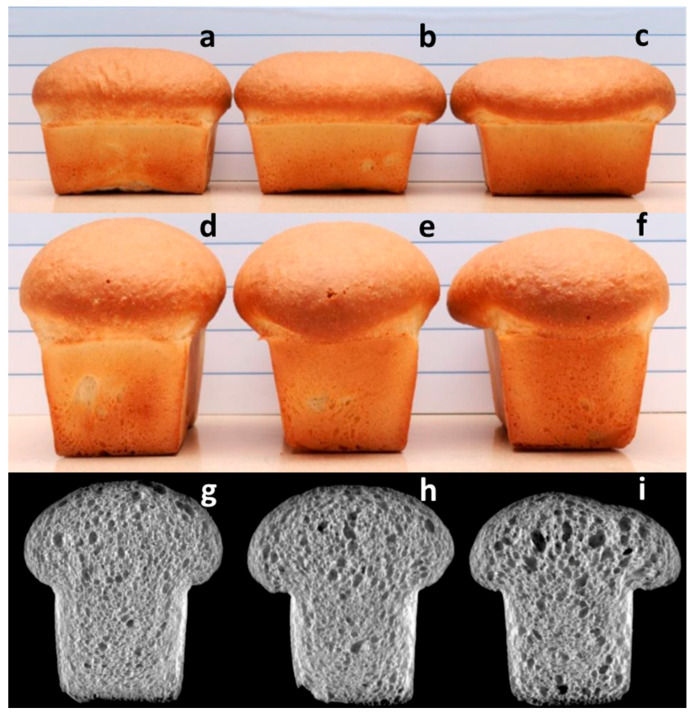
Bread loaves prepared from sound flour (**a**,**d**,**g**), 3% affected FDK flour (**b**,**e**,**h**) and 3% distorted FDK flour (**c**,**f**,**i**). Loaf volumes were comparable, however both FDK loaves exhibited loaf top collapse (top and middle rows) and contained a more open crumb structure with larger open cells. These deformities are indicative of protein damage.

Bread prepared with flour from blends spiked with 3% FDK-A (Figure 2b,e,h) and 3% FDK-D (Figure 2c,f,i) had similar loaf volume to sound flour (Figure 2a,d,g), but exhibited collapse of the loaf top (Figure 2a–f) and a more open cell structure in cross sections (Figure 2g–i), indicating the weakening of dough due to FDK protease. Loaf top ratios, calculated from loaf top measurements as described [28], were significantly lower in the FDK-A and FDK-D loaves (0.45 ± 0.01 SD and 0.44 ± <0.01 SD, respectively) than the sound loaf (0.51 ± 0.01 SD), indicating a loss of dough strength. This damage was apparent, as the FDK-A and FDK-D loaves had collapsed over the lip of the pans while the sound flour loaf maintained its loaf shape (not shown).

### 3.2. Ground Grain and Single Seed Protease Activity

**Figure 3:**
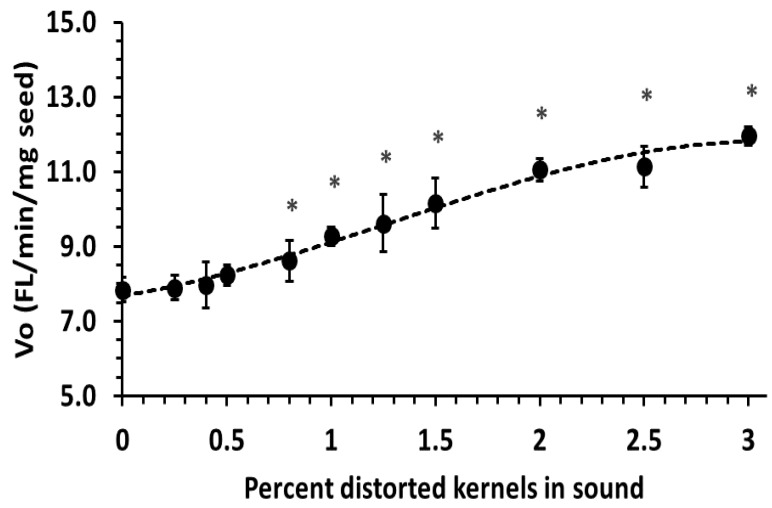
Protease activity, assayed with the fluorescent peptide substrate Mca-R-P-K-V-E-Nval-W-R-K(Dnp)-NH2, for ground seed samples of Fusarium damaged kernels (distorted) in sound (uninfected) base seed. Data points annotated with * are significantly higher than the 0% FDK level (two-tailed *t*-test, *α* = 0.05, *n* = 5).

Protease activity, assayed with the FRET-labeled peptide Mca-R-P-K-V-E-Nval-W-R-K(Dnp)-NH2 and expressed as initial reaction rates (fluorescence units per minute per mg seed) for FDK-D blends demonstrated a non-linear but positively correlated increase in activity with FDK content (Figure 3). Activity was significantly higher (two-tailed *t*-test, *α* = 0.05, *n* = 5) than sound base (0% FDK) at and above the 0.8% level.

**Figure 4:**
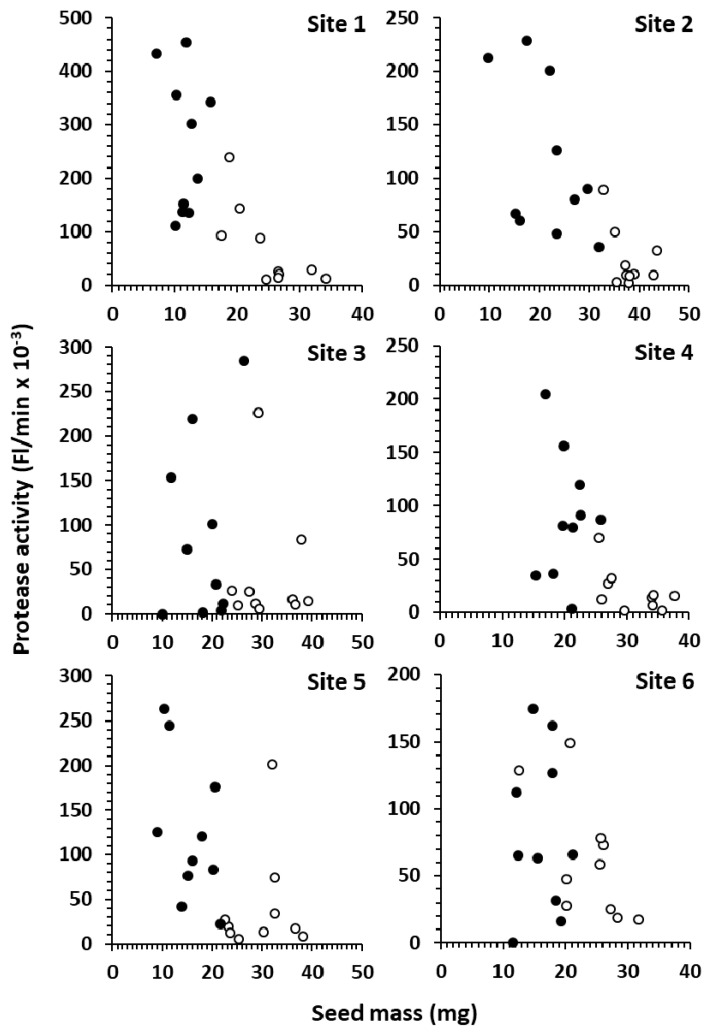
Protease activity from single affected (○) or distorted (●) FDK kernels in samples from six sites across Western Canada relative to seed mass. Activity varies widely between sites and among affected and distorted FDK.

FDK were hand-picked by trained inspectors from six producer samples across Western Canada and 10 seeds of FDK-A and FDK-D were analyzed for protease activity. Since reduced seed size was the definitive criteria for FDK-D, they were lighter than FDK-A (17.3 mg ± 5.4 mg SD and 29.8 mg ± 6.7 mg SD, respectively). The protease activity profiles differed from site to site (Figure 4): In sites 1, 2 and 4 there was a clear distinction between FDK-A and FDK-D in terms of both seed mass and activity, while in site 6 there was nearly a complete overlap.

### 3.3. Verification of Protease Specificity

Bovine beta-casein was subjected to digestion by enzyme extracts prepared from whole meal samples of sound base and sound base spiked with FDK-D. Due to the likely presence of multiple proteases and specificities, peptide mapping was performed with non-specific cleavage rules. Sequences were tabulated ± 4 amino acids for both the *N*- and *C*-termini of each identified peptide and expressed as the ratio (FDK-D/sound) for each position (Table 1). According to convention, sequence positions are numbered from the cleavage site starting from one, with prime (‘) notation used on the *C*-terminal side. Cleavage occurred eight-fold more frequently with lysine at P1, four-fold with isoleucine at P1, three-fold with tyrosine at P1 and five-fold more with tyrosine at P1′ in FDK-D digests (Table 1).

## 4. Discussion

While gluten strength is largely determined by the glutenin polymer size distribution and ratio of polymer to monomer (mostly gliadins), physical dough properties and baking performance depends on gluten strength and the amount of gluten proteins in a flour. Gluten functionality can be defined as the balance of the extensibility (stretchiness) and elasticity (springiness) of a dough, as the dough must be able to readily expand to accommodate the increasing sizes of gas cells during leavening but also maintain integrity without collapse. Excessive elasticity results in insufficient leavening as the gas pressure required to stretch the dough is too high, while excessive extensibility without sufficient strength results in rupture of gas cells leading to large voids within the loaf and collapse during baking. The extensigraph measures this balance of resistance (R_max_) and extensibility, and is a standard test for gluten functionality. A decrease in R_max_ generally correlates to a loss of dough strength and baking quality. The physical manifestation of the effect of *Fusarium* protease on baking quality is evident in loaves baked from sound flour versus 3% affected FDK and 3% distorted FDK (Figure 2). The combination of comparable loaf volume and partial collapse of the loaf top (Figure 2b) is indicative of protein degradation, in this case arising from *Fusarium* protease. Since *Fusarium* protease remains active through the dough making and proofing stages, processes with longer rest and proofing times are likely to result in greater loss of dough strength and loaf shape.

The extensigraph test is one of the most sensitive tests for measuring the viscoelastic properties of dough. The R_max_ (135 min) was significantly reduced from 445 BU to 382 BU at 1.5% FDK-A (Figure 1a). This can affect the dough handling properties during the bread-making process, even though loaf distortion was relatively small even at 3% FDK-A (Figure 2b,e,h). In general, an increase in R_max_ is expected with longer resting time, due to continued re-polymerization of the gluten network, demonstrated by the approximately 55 BU difference between 0% FDK results at 45 min and 135 min (Figure 1a). Superimposed on this gain is a loss of viscoelastic strength with increased FDK content arising from proteolytic damage to gluten; while R_max_ is generally higher at 135 min (Figure 1a) than at 45 min, the gain from the longer rest time of the 135 min test was essentially negated at 1.5% and 3% FDK-A and exceeded at 3% FDK-D. Expression of the data as a percent R_max_ reduction from 0% FDK for each series (Figure 1b) effectively removed the re-polymerization effect and revealed the protease-mediated functionality loss. In all cases, the relative R_max_ loss was greater at 135 min than at 45 min (Figure 1b), demonstrating that longer rest times resulted in a greater reduction in R_max_. This is consistent with previous reports, showing that in addition to damage during seed maturation, *Fusarium* protease damage occurs during fermentation [4,16]. The comparison of 45 and 135 min extensigraph data was effective at revealing the offsetting gluten strength loss due to protease from the expected gain from continued dough development (re-polymerization).

The protease assay was capable of detecting significantly elevated protease activity at 0.8% FDK-D (Figure 3), where extensigraph R_max_ was visibly affected (Figure 1a). The correlation of protease activity with decreased gluten strength, an indicator of baking quality, is consistent with previous reports relating *Fusarium* protease to reduced baking quality [3,11]. Although the assay time was 30 min, most samples progressed past the linear response region before this, so timed measurements and calculation of initial reaction rates from initial (linear) data were necessary. Digestion of beta-casein with enzyme extracts from FDK-D and sound seed showed that lysine at P1 (*N*-terminal to cleavage site) occurred eight-fold more with FDK-D extract than with sound extract, isoleucine at P1 four-fold more and at P1′ three-fold more, tyrosine at P1 three-fold more and arginine at P1′ five-fold more. This is consistent with previously identified subtilisin-like [20] and trypsin-like [21] proteases purified and characterized from *F. culmorum* and matching four potential cleavage sites in the artificial substrate Mca-R-P-K-V-E-Nval-W-R-K(Dnp)-NH2 by *Fusarium* proteases; *C*-terminal to both arginines and *N*-terminal to both lysines. To our knowledge, this is the first analysis of the specificity of the residual active *Fusarium* protease in flour after harvest, storage and milling into flour.

In Canada, wheat grading for Fusarium damage is based on FDK content, meaning FDK-M, FDK-A and FDK-D would all be counted equally for grading. Canadian bread wheat varieties predominantly belong to the CWRS class, with Fusarium damage (%FDK) limits of 0.3%, 0.8% and 1.5% for No. 1, No. 2 and No. 3 CWRS grades, respectively [30]. The use of FDK-D presents a worst-case scenario, as real producer samples would likely contain a range of less infected kernels and FDK-D can be partially removed by commercial cleaning due to their lower density and thin shape. Since extensigraph R_max_ and protease activity were significantly changed at and above 0.8% FDK-D (Figure 1 and Figure 3), there should be very low risk to dough handling properties, and baking quality provided *Fusarium* damage, as expressed as FDK%, does not exceed the No. 2 CWRS limit. The development of a limit-test for *Fusarium* protease may be useful for grade improvements to “average out” damage types by blending sound and damaged seed lots in cases where DON levels meet the specifications. 

Data from single seed analysis of FDK-A and FDK-D from six producer sites across Western Canada demonstrate the complex problem that *Fusarium* protease presents. Raw activities were generally higher in FDK-D than FDK-A across sites, and they formed distinct groups in sites 1, 2 and 4, but were co-mingled in site 6 (Figure 4). Since FDK-D seeds are light weight and thin by definition, they can mostly be removed by commercial cleaning. This may result in a dramatic reduction in total protease for many samples due to the higher activity in FDK-D; however, sites 3, 5 and 6 (Figure 4) contain FDK-A seeds (which cannot be effectively removed by commercial cleaning) with activities similar to FDK-D seeds. This suggests that remediation by cleaning may be highly variable and sample-specific. The highest activity in the blended samples (3% FDA-D) was 11.6 Fl/min/mg seed (Figure 2), compared to activities of FDK-A and FDK-D (normalized to seed mass) spanned 53.0–12,700 Fl/min/mg seed and 0 (not detected) to 61,500 Fl/min/mg seed, respectively. The incredibly high potential activity of individual seeds, combined with a wide range of measured activities, indicates that the extent of infection (% FDK) and the severity of infection (types of FDK) both play a role in defining the total protease in a sample.

While protease activity from *Fusarium* infection reduces gluten strength by partial digestion of polymeric gluten, not all samples are functionally impacted to the same extent. Flours from varieties with higher intrinsic gluten strength may demonstrate a reduction in dough elasticity, but the loss of their dough handling properties and baking performance was not as much as for flours from weaker varieties [31]. Kreuzberger et al. [10] have presented evidence that the potential damage from Fusarium protease is minimal for samples under the EU DON limit. Our results indicate that the visual grading system in Canada equally protects quality, as there was no significant reduction in extensigraph R_max_ below 0.8% FDK; the current limit for the No. 2 CWRS grade [30]. It may appear that the potential quality reduction from *Fusarium* protease can be controlled, since variability in gluten strength normally occurs for a variety of reasons and is routinely compensated for by wheat blending or changes to milling process or baking formulations. However, changes in process or reformulation is time consuming and costly for food manufacturers. In addition, inconsistency of raw material quality is a major concern for millers and bakers. Other options, such as using DON as a proxy for potential protease damage, also need to be regularly and regionally validated due to the prevalence of DON non-producers, such as *F. avenaceum*. Ultimately, a thorough knowledge of the variability of protease expression between fungal species and strains within a growing region, in response to environmental stresses and to wheat varieties is valuable to fully validate any grading or quality control system.

## Figures and Tables

**Table 1:** Heat map (red = low, green = elevated) showing the relative frequency of amino acids near cleavage sites of bovine beta-casein digested with enzyme extracts from distorted *Fusarium*-damaged kernels (FDK-D) relative to clean (uninfected) seed. Elevated numbers mean the specified amino acid occurred at a position in more peptides in the FDK-D digest than base seed digest, i.e., lysine was eight times more often at p1 in the FDK-D digest.

	**P4**	**P3**	**P2**	**P1**	Cleavage site	**P1′**	**P2′**	**P3′**	**P4′**
A	1	1	1	1		1	1	0	2
C	-----	-----	-----	-----		-----	-----	-----	-----
D	1	0	1	0		1	2	2	1
E	1	1	1	1		1	1	1	1
F	2	1	1	1		1	0	1	3
G	3	3	1	2		1	2	2	2
H	0	1	0	1		1	1	0	4
I	2	2	2	4		3	1	1	2
K	1	0	1	8		1	4	6	1
L	1	1	1	1		1	1	1	1
M	2	0	0	1		1	1	14	0
N	1	2	1	1		0	1	0	2
P	1	1	2	1		1	1	1	1
Q	1	1	1	1		1	1	1	1
R	1	1	1	1		5	-----	1	1
S	1	1	1	1		1	1	1	1
T	0	1	1	1		1	1	1	1
V	1	1	1	1		1	2	1	2
W	0	0	-----	0		1	-----	0	8
Y	0	8	2	3		1	1	1	2

## Data Availability

The data presented in this study are openly available in Mendeley Data at doi:10.17632/93pb9nv6hy.1 [32].
